# Beyond sightseeing: How can tourism affect public/global health in modern society?

**DOI:** 10.7189/jogh.12.03035

**Published:** 2022-06-28

**Authors:** Jun Wen, Metin Kozak, Yangyang Jiang

**Affiliations:** 1 School of Business and Law, Edith Cowan University, Joondalup, Australia; 2 Department of Advertising, School of Communication, Kadir Has University, Istanbul, Turkey; 3 Nottingham University Business School China, University of Nottingham Ningbo China, Ningbo, China

## A REFLECTION OF TOURISM RESEARCH

Tourism was historically regarded as a practical and business-oriented domain rather than as a research discipline [1,2]. Ontologically, tourism was seen as a field in which to apply theory and as a means of fulfilling needs related to leisure, pleasure, and social health. Little effort has been made to uncover its more nuanced meanings. In 2006, John Tribe, a leading tourism scholar, published a commentary challenging the truth of tourism research and described a complex phenomenon in which the construct’s psychological, philosophical, and social dimensions/values were not well understood [3]. He also suggested that much of the research published on tourism was influenced by key factors related to the research authors [3].

Recent decades have witnessed extensive developments in tourism studies within the social sciences, which have been recognised as a strong research context [4,5]. Scholars first borrowed information from external disciplines (eg, geography, sociology, economics, business, environment, among others) and then exported knowledge to other fields, eg, economics, geography, environment, culture, and business, among others [1]. Despite the now robust pool of tourism research, two salient questions continue to puzzle tourism academics: 1) What knowledge can tourism produce and/or sell (eg, experiences, memories) and 2) What role does tourism play in modern society, especially regarding collaboration with other fields (eg, public health and psychology)? This viewpoint highlights the connection between tourism and public/global health and provides evidence of tourism’s updated role in modern society.

## THE NEXUS BETWEEN TOURISM AND PUBLIC HEALTH

Prior to the COVID-19 outbreak, tourism was the world’s largest and fastest-growing industry. This industry was also one of the most sharply affected by travel restrictions and pandemic prevention policies [6]. In addition, wars, diseases, natural disasters, political turmoil, and other catastrophes have moulded the international landscape along with individuals’ behaviour and perceptions. The COVID-19 pandemic brought increased attention to the possible nexus between tourism and public health. This intersection offers an opportunity to contemplate the nature of tourism and the roles it can play in public health; for example, travellers with physical and/or psychological disorders (ie, vulnerable populations) have been largely neglected in tourism literature and industry compared with typical tourists [7].

Tourism has been found to boost tourists’ physical, psychological, and social well-being [8,9]. It is, therefore, necessary to scrutinize tourism’s place in global health, not only for so-called normal (healthy) groups, but also for vulnerable groups, to explore how to enhance health via related activities. For people living in war zones (eg, the area of the 2022 Russia-Ukraine conflict) who are suffering physical and emotional consequences, tourism may foster mental health and psychic healing by developing specific programs in later periods, so-called “solidarity tourism”. In relation to hospitality, for example – as Poland welcomed the Ukrainian people to stay peacefully within its borders, some people hired hotels to host the Ukrainians. In Turkey, many hotels in Izmir opened their doors to people negatively affected by the earthquake on October 30, 2020. Also, during the early weeks of the pandemic in Turkey, hoteliers provided the health staff with free accommodation and food, as they were unable to return home for a rest after work due to the fierce restrictions.

**Figure:**
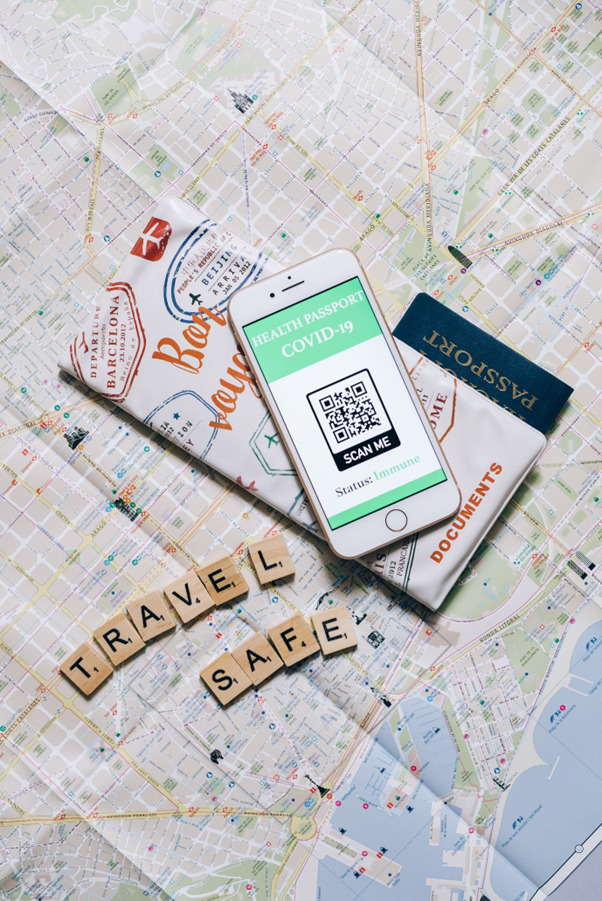
Photo: Source: https://www.pexels.com/photo/close-up-shot-of-scrabble-tiles-on-a-map-8372640/. No permission needed.

From an epistemological perspective, integrating distinct scientific disciplines to resolve complex real-world problems is challenging [10]. This circumstance complicates the task of unravelling empirical observations and subjective accounts [11] in order to conduct in-depth empirical work on tourism. Interdisciplinary studies may contribute to the accumulation of such information: experts from various disciplines can bring epistemic resources, such as concepts, models, theories, and data, to strengthen tourism-related knowledge. For instance, public health can infuse tourism research with a fresh perspective, enabling scholars to generate novel insight into individual wellness, public health, and global health. Bauer [12] argued that travellers with disabilities or those otherwise unable to partake in tourism have been especially neglected, hence the call for more studies to understand their needs.

Based on medical science literature on disability, Bauer [12] explored these travellers’ motivations, barriers, and experiences. His findings led to useful information for both health professionals and tourism and hospitality staff to more effectively serve this group of travellers. Connell and Page [7] noted that complex and sometimes invisible health conditions, such as dementia and autism, pose challenges for the tourism industry. These and similar topics necessitate a shift in tourism business strategies to cater to affected tourists and their caregivers. The research agenda put forward by Connell and Page [7] is in line with work conducted by Page, Innes, and Cutler [13], which focused on the growing global issue of dementia and solicited tourism service providers’ opinions about obstacles and opportunities to develop dementia-friendly tourism destinations. Connell and Page [14-17] subsequently examined tourism destinations’ readiness to offer dementia-friendly tourist experiences, building on interdisciplinary studies from the medical and social sciences.

## FUTURE RESEARCH ON TOURISM AND/OR GLOBAL PUBLIC HEALTH

Tourism research is inherently multidisciplinary, having its roots in established fields such as economics, sociology, psychology, anthropology, and others [3,4]. We have highlighted a nexus between tourism and public health/global health (eg, the pandemic and vulnerable travellers) and provided evidence of tourism’s updated roles in modern society (eg, healing after a war). After years of learning from other fields, tourism scholars do have a product to sell and a story to share about tourism’s place in people’s lives, by combining knowledge from disciplines such as public health, psychology, and epidemiology [18-20]. After enduring the COVID-19 pandemic and now pondering ways that tourism can support people living in war zones (eg, the Russia-Ukraine conflict and the potential for solidarity tourism), more careful deliberation of the question “Is tourism associated with global and/or public health?” will be appreciated to promote tourism research with a wider scope.
